# Concurrent and Angle-Trajectory Validity and Intra-Trial Reliability of a Novel Multi-View Image-Based Motion Analysis System

**DOI:** 10.5114/jhk/159587

**Published:** 2023-01-20

**Authors:** Namgi Lee, Junghoon Ahn, Wootaek Lim

**Affiliations:** 1 Department of Physical Therapy, Kwangju Women’s University, Gwangju, Republic of Korea.; 2 Department of Health Science, Graduate School, Korea University, Seoul, Republic of Korea.; 3 Department of Physical Therapy, Woosong University, Daejeon, Republic of Korea.; 4 Woosong Institute of Rehabilitation Science, Daejeon, Republic of Korea.

**Keywords:** kinematics, motion capture system, motion analysis system, reliability, validity

## Abstract

Sports-related injuries are the most common in the lower extremities among physical regions. To evaluate impaired functional performance in sports training facilities and sports, a marker-less motion analysis system that can measure joint kinematics in bright indoor and outdoor environments is required. The aim of this study was to establish the concurrent and angle-trajectory validity and intra-trial reliability of a novel multi-view image-based motion analysis system with marker-less pose estimation during lower extremity tasks in healthy young men. Ten healthy young men participated voluntarily in this study. The hip and knee joint angles were collected using a multi-view image-based motion analysis system (marker-less) and a Vicon motion capture system (with markers) during the lower extremity tasks. Intraclass correlation coefficient (ICC) analyses were used to identify the concurrent and angle-trajectory validity and intra-trial reliability of the multi-view image-based motion analysis system. In the concurrent validity, the correlation analysis revealed that the ICC3, k values on the hip and knee flexions during knee bending in sitting, standing, and squat movements were from 0.747 to 0.936 between the two systems. In particular, the angle-trajectory validity was very high (ICC3, 1 = 0.859–0.998), indicating a high agreement between the two systems. The intra-trial reliability of each system was excellent (ICC3, 1 = 0.773–0.974), reflecting high reproducibility. We suggest that this novel marker-less motion analysis system is highly accurate and reliable for measuring joint kinematics of the lower extremities during the rehabilitation process and monitoring sports performance of athletes in training facilities.

## Introduction

An average annual estimate of 8.6 million sports-related injuries was reported between 2011 and 2014 in the USA, which represents an age-adjusted rate of 34.1 per 1,000 persons. The sports-related injuries reported more than one-half of the injury episodes in males (61.3%) and persons aged 5–24 years (64.9%), owing to the fact that the types of activities differed by sex and age groups. Physical regions injured while participating in sports activities included lower extremities (42.0%), upper extremities (30.3%), and the head and the neck (16.4%) (Sheu et al., 2016). Common lower extremity injuries include strains, sprains, tendon rupture, dislocation, and fractures that occur during team ball sports, such as basketball, soccer, volleyball, and field hockey (Meehan and Mannix, 2013; Sheu et al., 2016). A pathologic problem at the hip joint can cause immediate gait abnormalities, resulting in chronic pain and early degeneration in the hip joint. Additionally, knee injuries are the most common sports-related injuries associated with pain, physical weakening, hindering sports participation, depression, and early-onset osteoarthritis. One-half of sports- related injury episodes results in emergency department visits or hospitalizations (Sheu et al., 2016). To return to sports after lower extremity injuries, the rehabilitation of the injured athlete is managed by sports physicians and physiotherapists, coaches, and athletic trainers through assessment of lower extremity function. Standardized functional testing is used to compare functional performance data of pre-injury or normative data of healthy athletes (Haitz et al., 2014).

Three-dimensional (3D) motion capture with a marker-based tracking system (e.g., Vicon motion capture system) is known as the gold standard to assess functional performance, such as joint analysis, in both clinical and sports settings (Zhou and Hu, 2008). The 3D motion analysis is considered a key objective indicator in planning treatment interventions and monitoring treatment efficiency (Gajdosik and Bohannon, 1987). A number of professionals, including physicians and physiotherapists, coaches, and athletic trainers have been performing objective outcome-based care, concerning for example joint angles, and thus, the use of valid and reliable instruments to measure the joint angle is imperative. The joint angle measures represent the index of change in sports functional performance or the evaluation outcome value to therapeutic interventions during rehabilitation programs (García-Rubio et al., 2019; Lim, 2021; Oh et al., 2019). To achieve accurate and reliable results, highly skilled and well-trained operators are required to calibrate and run the 3D motion capture system; thus, they are not easily available to all professionals (Krause et al., 2015). Although the 3D motion capture system is the most valid and reliable measure, it is expensive, and requires a set-up environment which has limited feasibility in most rehabilitation and sports training facilities (Bahadori et al., 2019). In particular, it is difficult to measure the joint angle in bright indoor and outdoor areas, such as sports training facilities and fields, because the camera is equipped with an infrared strobe to emit a light signal and collect the reflected signal from the markers.

To overcome this challenge, a multi-view image-based motion analysis system has been developed that reliably measures the joint kinematics in bright indoor and outdoor conditions, regardless of obstacles (e.g., other functional measure equipment) near the testing area. That is, this system has the capability to achieve motion tracking with marker-less pose estimation based on image analysis technology in a space without environmental restrictions. Although marker-less motion capture technology (commonly images) has gained an increasing attention in biomechanics, there is a limited number of studies for comparing the difference between the marker-less motion capture technique and marker-based motion capture technique (Ceseracciu et al., 2009, 2014). Therefore, the aim of this study was to establish the concurrent and angle-trajectory validity of a novel multi-view image-based motion analysis system with markerless pose estimation through hip and knee joint angle measurements by comparing them with joint angle data obtained using a Vicon motion capture system with markers. In addition, this study was conducted to determine the intra-trial reliability of the multi-view image-based motion analysis system and Vicon motion capture system in healthy young men.

## Methods

### Participants

In this study, ten health young men (age = 25.4 ± 2.0 years, body height = 174.4 ± 5.0 cm, body mass = 68.9 ± 6.8 kg) who could perform the experimental tasks, such as knee bending in sitting and standing positions and deep squat movements, accurately and consistently, participated voluntarily. Participants were excluded if they had a current or past history of neurological, musculoskeletal, or cognitive system disorders. Prior to the commencement of the study, participants were informed regarding the purpose and procedures of the study and signed an informed consent form. The experimental protocol followed the Declaration of Helsinki and was approved by the Institutional Review Board of the Woosong University before its execution.

### Measures

#### Multi-View Image-Based Motion Analysis System

**Figure 1:**
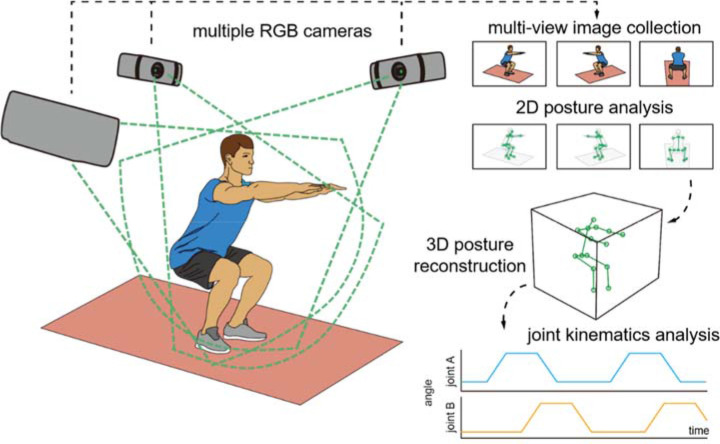
Multi-view image-based motion analysis system.

A multi-view image collection system consisting of four red-green-blue (RGB) cameras (4DEYE, SYM healthcare lnc., Seoul, Republic of Korea) was used to capture participants’ posture at 30 Hz from four different directions (Figure 1). After image collection, the angles of the hip and knee joints were analyzed using a custom analysis program developed based on the open source image analysis libraries; OpenCV (Bradski and Kaehler, 2000) and OpenPose (Cao et al., 2021). Specifically, OpenPose software estimated the two-dimensional positions of seven physical keypoints, including the neck, the left shoulder, the right shoulder, the mid hip, the right hip, the knee, and the ankle, in each of the four images simultaneously captured by the four cameras. Then, OpenCV software reconstructed the three-dimensional position of each keypoint from the four different two-dimensional positions of the keypoint based on information on the relative position and orientation of the cameras.

Hip flexion/extension was described as the angle of the femoral shaft relative to the trunk, while knee flexion/extension was described as the angle between the femoral and tibial shafts. First, the trunk coordinates were obtained as follows: the Z-axis of the trunk was defined as a vector pointing to the neck from the mid-hip. The X-axis was defined as a vector normal to the plane consisting of the left shoulder, the right shoulder, and the mid hip. The Y-axis was a vector orthogonal to the Zand X-axes. Subsequently, the femoral and tibial shaft vectors were defined as vectors pointing the knee from the right hip and the ankle from the knee, respectively. To quantify the hip flexion in the three-dimensional space regardless of the plane of hip flexion, the hip flexion angle was calculated as the angle between the negative Z-axis of the trunk coordinate and the femoral shaft vector. As the leg raised, the hip flexion angle increased from 0° (i.e., anatomical neutral posture) to 180°. Finally, the calculated joint angles were interpolated to match the data length with the data collected at 100 Hz using the Vicon motion capture system.

#### Vicon Motion Capture System

A Vicon motion capture system (MX T series, Oxford Metrics, Ltd., Oxford, UK) has proprietary hardware to capture the coordinates of the positioning points using eight infrared (IR) cameras. This system also requires retro-reflective markers to the emitted IR light signal from the IR strobe of each camera. Four markers (14-mm in diameter) were attached to the trunk and lower extremity landmarks, including the seventh cervical vertebrae (C7), the eighth thoracic vertebrae (T8), the jugular notch, and the xiphoid process of the sternum. Two cross-shaped clusters consisting of four markers were attached to the thigh and the shank. One axis of the cross was aligned to the femoral or tibial shaft. Each camera captured the three-dimensional locations of all markers at 100 Hz. Joint angles were calculated in a similar manner as the analysis based on the multi-view motion capture system, however, the trunk coordinate, the femoral shaft, and the tibial shaft vector were defined differently using the positioning points of each marker. The trunk coordinate was obtained as described by Wu and colleague’s methods (Wu et al., 2005). The femoral and tibial shaft vectors were obtained using a cross-shaped cluster. The joint angle analysis was conducted using MATLAB R2018A (The Mathworks, Inc., Natick, MA, USA).

### Procedures

The lower extremity tasks consisted of knee bending in sitting and standing (open kinematic chain) and squat movements (closed kinematic chain). First, to perform the knee bending while sitting, the starting posture was that participants sat on a chair without a back and arm rest, and maintained 90° of knee flexion. Participants performed full extension of the knee joint and repositioned them toward the starting posture. Second, for knee bending while standing, participants maintained standing with full knee extension (starting posture), and then they performed knee bending up to approximately 90° flexion. Finally, to perform the squat movement, the starting posture was that the feet were located shoulder width apart with arms stretched out anteriorly to the body and parallel to the floor. Participants performed a deep squat and then moved towards the starting posture (Harsted et al., 2019). Each lower extremity task was performed in five trials with a 5-s rest interval between each trial, and the rest interval between experimental tasks was three to five minutes in this study. During the lower extremity tasks, the joint angle data on hip and knee flexion were collected and processed, and each trial data and average data of trials were used for further analysis.

### Statistical Analysis

Descriptive statistics included mean and standard deviations. Intra-class correlation coefficients (ICCs) and 95% confidence intervals (CIs) were used for the analysis of concurrent and angle-trajectory validity (ICC3, k) between the novel multi-view image-based motion analysis system (marker-less) and the Vicon motion capture system (with markers). ICC analysis was used to assess the intra-trial reliability (ICC3, 1) of each motion analysis system. ICC values can be interpreted as follows: ICC< 0.50 (poor), 0.50–0.75 (moderate), 0.76–0.90 (good), and > 0.90 (high). In addition, the coefficient of variation (CV), standard error of measurement (SEM), and minimal detectable change (MDC) were calculated to find absolute reliability (Overend et al., 2010; Weir, 2005). The CV for method error was calculated as follows: CV = 100 × (2 × (SDd /√2)/(X1 + X2)); SDd = standard deviation (SD) of the differences between two measures, X1 and X2 = each mean of the two measures (Portney and Watkins, 2009). The SEM was calculated as follows: SEM = SD × √(1 – ICC) to provide a measure of variability and was used to calculate the MDC. Finally, the MDC represents a statistical estimate of the smallest amount of change to provide confidence that a change was not the result of subject variability or measurement error, and was calculated as follows: MDC = z-score (95% CI) × SEM × √2 (Haley and FragalaPinkham, 2006). The level of significance was set at *p* < 0.05. All statistical analyses were performed using SPSS for Windows (version 18.0; SPSS Inc., Chicago, IL, USA) and Microsoft Excel 2019 (Microsoft Inc., Redmond, WA, USA).

## Results

### Validity

The concurrent validity of the novel multi-view image-based motion analysis system (marker-less) was determined by comparing the Vicon motion capture system (with markers) through hip and knee flexion angles during lower extremity tasks, as shown in Table 1. Correlation analysis revealed that the ICC3, k values on the knee flexions in sitting and standing were 0.747 (95% CI = −0.017–0.937, CV = 5.80%) and 0.780 (95% CI = 0.116–0.945, CV = 5.23%), respectively. The hip and knee flexions during squat movement showed high validity (ICC3, k = 0.902 and 0.936; 95% CI = 0.606–0.976 and 0.743–0.984; CV = 4.11 and 4.10%, respectively) of the multi-view image-based motion analysis system (Table 1).

**Table 1:** Concurrent validity between the novel multi-view image-based motion analysis system and the Vicon motion capture system.

Motion	Measurement	Mean ± SD (°)	ICC _(3, *k*)_	95% CI	CV (%)
Knee flexion (sitting)	MI-MAS VMCS	89.94 ± 10.01 82.87 ± 11.68	0.747*	-0.017–0.937	5.80
Knee flexion (standing)	MI-MAS VMCS	111.88 ± 5.45 101.65 ± 11.99	0.780†	0.116–0.945	5.23
Hip flexion (squatting)	MI-MAS VMCS	105.57 ± 14.75 103.00 ± 13.41	0.902†	0.606–0.976	4.11
Knee flexion (squatting)	MI-MAS VMCS	104.11 ± 16.15 116.73 ± 15.83	0.936†	0.743–0.984	4.10

MI-MAS 104.11 ± 16.15 0.936† 0.743–0.984 4.10 VMCS 116.73 ± 15.83 MI-MAS, multi-view image-based motion analysis system without markers; VMCS, Vicon motion capture system with markers; SD, standard deviation; ICC, intraclass correlation coefficient based on the model (3) and type (the mean of k raters/measurements); * p < 0.05; † p < 0.01; CI, confidence interval; CV, coefficient of variation.

**Figure 2:**
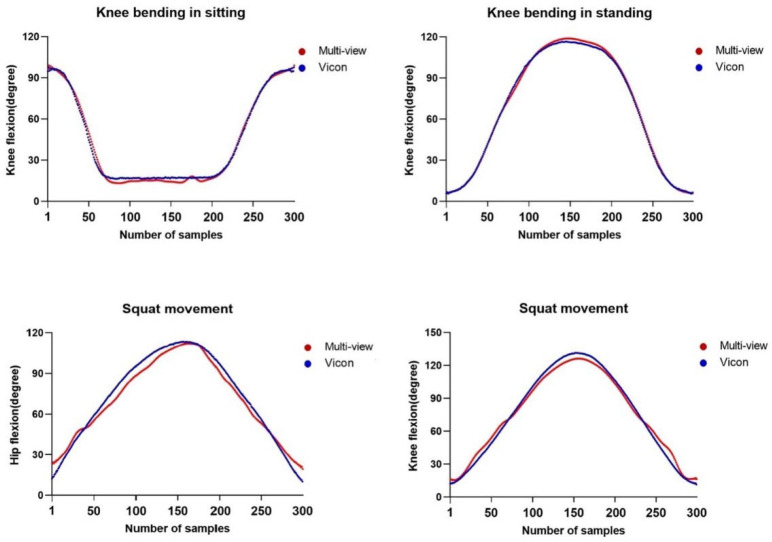
Representative joint angle graphs indicating the angle-trajectory validity of the multi-view image-based motion analysis system.

The angle-trajectory validity of the hip and knee joint angles was represented by comparing one trial data of each system through full range of motion, and the validity data of each participant are presented as shown in Table 2. Correlation analysis identified that ICC3, 1 values of each participant on the knee flexion in sitting and standing were very high (ICC3, 1 = 0.938–0.998 and 0.859–0.998, respectively). ICC3, 1 values for the hip and knee flexion during squat movement were 0.970–0.995 and 0.926–0.994, respectively (Table 2). The representative joint angle graphs to reveal the angle-trajectory validity of the multi-view image-based motion analysis system are shown in Figure 2.

**Table 2:** Angle-trajectory validity between the novel multi-view image-based motion analysis system and the Vicon motion capture system.

	Knee flexion (sitting)		Knee flexion (standing)		Hip flexion (squatting)		Knee flexion (squatting)	
Subject	ICC _(3, 1)_	95% CI	ICC _(3, 1)_	95% CI	ICC _(3, 1)_	95% CI	ICC _(3, 1)_	95% CI
1	0.988†	0.984– 0.990	0.998†	0.998–0.998	0.991†	0.989– 0.993	0.988†	0.984– 0.990
2	0.938†	0.923– 0.950	0.998†	0.998–0.999	0.994†	0.993– 0.996	0.994†	0.992– 0.995
3	0.991†	0.988– 0.993	0.995†	0.994–0.996	0.975†	0.969– 0.980	0.968†	0.960– 0.974
4	0.988†	0.986– 0.991	0.971†	0.963–0.976	0.984†	0.980– 0.987	0.985†	0.982– 0.988
5	0.955†	0.944– 0.964	0.992†	0.990–0.994	0.993†	0.991– 0.994	0.946†	0.932– 0.956
6	0.948†	0.935– 0.958	0.990†	0.987–0.992	0.970†	0.963– 0.976	0.953†	0.941– 0.962
7	0.989†	0.986– 0.991	0.993†	0.991–0.994	0.995†	0.994– 0.996	0.986†	0.982– 0.989
8	0.985†	0.981– 0.988	0.993†	0.991–0.994	0.987†	0.984– 0.990	0.926†	0.908– 0.941
9	0.998†	0.998– 0.999	0.990†	0.988–0.992	0.986†	0.982– 0.989	0.978†	0.972– 0.982
10	0.950†	0.938– 0.960	0.859†	0.826–0.886	0.992†	0.991– 0.994	0.966†	0.957– 0.973

ICC, intraclass correlation coefficient based on the model (3) and type (single measurement); † p < 0.01; CI, confidence interval.

### Reliability

The intra-trial reliability was determined by repeated measures of the novel multi-view image-based motion analysis system (marker-less) and the Vicon motion capture system (with makers), and is presented in Table 3. Comparing ICC3, 1 values for knee flexion while sitting, the value obtained using the multi-view image-based motion analysis system was 0.918 (95% CI = 0.705– 0.979, CV = 2.83 %, SEM = 4.59, MDC = 12.71), while this obtained using the Vicon motion capture system was 0.969 (95% CI = 0.879–0.992, CV = 1.78 %, SEM = 4.72, MDC = 13.07). The ICC3, 1 values for knee flexion while standing were 0.773 (95% CI = 0.321–0.938, CV = 2.21 %, SEM = 3.66, MDC = 10.14) and 0.879 (95% CI = 0.587–0.968, CV = 3.14 %, SEM = 4.31, MDC = 11.96), indicating good reliability. The ICC3, 1 values for hip flexion during squat movement showed high reliability (ICC3, 1 = 0.887 and 0.974; 95% CI = 0.611–0.971 and 0.898–0.993; CV = 3.13% and 1.43%; SEM = 5.10 and 2.17; MDC = 14.15 and 6.02) using both systems. Finally, the ICC3, 1 values for knee flexion during squat movement showed high reliability in both the multi-view image-based motion analysis system (ICC3, 1 = 0.908, 95% CI = 0.673–0.976, CV = 1.82 %, SEM = 4.99, MDC = 13.82) and the Vicon motion capture system (ICC3, 1 = 0.970, 95% CI = 0.885– 0.992, CV = 1.20 %, SEM = 2.76, MDC = 7.65) (Table 3).

**Table 3:** Intra-trial reliability of the novel multi-view image-based motion analysis system and the Vicon motion capture system.

Motion	Measurement	Mean±SD (°) (Test 1)	Mean±SD (°) (Test 2)	ICC _(3, 1)_	95% CI
Knee flexion	MI-MAS	88.64 ± 9.52	91.23 ± 10.89	0.918†	0.705–0.979
(sitting)	VMCS	81.94 ± 11.04	83.80 ± 12.46	0.969†	0.879–0.992
Knee flexion	MI-MAS	110.99 ± 6.94	111.65 ± 8.42	0.773†	0.321–0.938
(standing)	VMCS	99.30 ± 11.99	101.64 ± 12.81	0.879†	0.587–0.968
Hip flexion	MI-MAS	104.16 ± 15.12	106.99 ± 15.24	0.887†	0.611–0.971
(squatting)	VMCS	102.17 ± 14.30	103.84 ± 12.65	0.974†	0.898–0.993
Knee flexion	MI-MAS	103.65 ± 18.23	104.57 ± 14.65	0.908†	0.673–0.976
(squatting)	VMCS	116.76 ± 16.63	116.70 ± 15.24	0.970†	0.885–0.992

MI-MAS, multi-view image-based motion analysis system without markers; VMCS, Vicon motion capture system with markers; SD, standard deviation; ICC, intraclass correlation coefficient based on the model (3) and type (single measurement); † p < 0.01; CI, confidence interval.

## Discussion

The aim of this study was to determine the concurrent and angle-trajectory validity as well as intra-trial reliability of the proposed multi-view image-based motion analysis system during lower extremity tasks in healthy young men. The results demonstrated that the novel multi-view image-based motion analysis system with marker-less pose estimation had high concurrent validity (ICC3, *k* = 0.747 to 0.936) when compared with hip and knee joint angles captured by the Vicon motion capture system with markers, as well as excellent reliability (ICC3, 1 = 0.773 to 0.974) when measured repeatedly. In particular, the angle-trajectory validity between these systems was very high (ICC3, 1 = 0.859 to 0.998) in measuring joint angles during lower extremity tasks, and it was revealed in all participants. We suggest that this novel marker-less motion analysis system is highly accurate and reliable for the measurement of joint angles or kinematics during human movement.

This study supports previous studies conducted on healthy young men and preschool children, which investigated the concurrent validity and reliability of multi-view image-based motion capture systems determined by comparing the Vicon motion capture system through kinematics of the upper and lower extremities (Cai et al., 2019; Harsted et al., 2019). Cai et al. (2019) investigated the concurrent validity and test-retest reliability of a Kinect V2 system based on 2D depth images during four upper limb tasks (hand to the contralateral shoulder, hand to the mouth, combing hair, and hand to the back pocket) in ten healthy men. The Kinect V2-based upper limb functional assessment system had high concurrent validity (Pearson’s *r* correlation, *r* = 0.74 to 0.99) and test-retest reliability (*r* = 0.70 to 0.96) of the range of motion in upper limb tasks (Cai et al., 2019). In another study, lower extremity kinematics data on squat and standing broad jump movements between the Captury based on a passive vision system and Vicon motion analysis system were compared in 14 preschool children. It was revealed that the repeated measures correlations (means concurrent validity of The Captury) on hip and knee flexion during squats and jumps ranged from 0.73 to 0.99 (Harsted et al., 2019). In addition, Ceseracciu et al. (2014) compared marker-less and marker-based motion capture technologies through kinematic gait data, and demonstrated that sagittal plane kinematics were estimated better than on the frontal and transverse planes in the hip, knee, and ankle joints.

3D motion capture systems with markers or trackers, such as the Xsens MVN BIOMECH system (Xsens Technologies B.V., The Netherlands) and a 3D motion analyzer (Shimano Dynamics Lab, Sittard, Netherland), also showed high validity and reliability when compared with kinematic data from the Vicon motion capture system (Al-Amri et al., 2018; Bouillod et al., 2016). Bouillod et al. (2016) highlighted the importance of marker placement for comparative statistical analysis between the two motion capture systems, and explained that differences observed between the systems were related to the displacement of the 3D motion analyzer markers during dynamic measurements (Bouillod et al., 2016). These marker-based 3D motion captures suffer from well-known shortcomings including obtrusion, expense, data errors owing to damage to the marker trajectories, long set-up times, requirement of operating skills, and the lack of ability to capture the dynamic motion of subjects in normal clothing (Krause et al., 2015; Xu, 2019). In contrast, the multi-view image-based motion capture system performs well in less controlled indoor settings or outdoors, and has advantages, such as low cost and no specific preparation of the subject (Elhayek et al., 2015; Xu, 2019). Therefore, many researchers have gained interest in multi-view image-based motion capture systems (Ceseracciu et al., 2014). To our knowledge, this study is the first attempt to investigate the angle-trajectory validity of a multi-view image-based motion analysis system without markers through lower extremity kinematic measures. Since this novel system is based on multi-view images from various perspectives, 3D motion analysis is possible. Moreover, regardless of the light intensity, such as an infrared strobe or LED marker, the joint kinematic data could be collected to evaluate the intervention effects during the rehabilitation process and monitor the sports performance of athletes in bright indoor and outdoor training facilities and sports fields.

Although this study revealed meaningful findings, certain limitations should be considered. First, the lower-extremity kinematics of this study only included sagittal plane motions of hip and knee flexion/extension, owing to the importance of hip and knee flexion-extension moments in sports injuries. It has been theorized that impaired strength in the sagittal plane musculature limits the amount of knee and hip flexion during dynamic tasks, and consequently, causes a greater dependence on passive limits in the frontal plane, such as ligaments, to decelerate the body's center of mass. For example, increased frontal plane loading at the knee is important because knee valgus angles and adductor moments are known as predictive factors of anterior cruciate ligament (ACL) injury. It has been reported in the literature that an ACL injury group exhibit a significantly greater hip flexion moments compared with an uninjured group (DeMorat et al., 2004; Weinhandl et al., 2014). This shows strong evidence demonstrating that sagittal plane factors contribute to the mechanism of the ACL injury. Further studies should investigate the upper or lower extremity kinematics of the sagittal, frontal, and horizontal planes during clinically relevant functional activities or various dynamic and fast sports performances. Second, studies analyzing joint kinematics on representative sports performances are also required in outdoor or sports fields because the data in this study were only collected in bright indoor environments. Finally, the current findings cannot be generalized to the sagittal plane kinematics of lower extremity motions, which may indicate the need for a large sample size in healthy adults or athletes.

This study investigated the concurrent and angle-trajectory validity and intra-trial reliability of a novel multi-view image-based motion analysis system. The findings of this study revealed good to high correlations in hip and knee flexion during lower extremity tasks between the multi-view image-based motion analysis system and Vicon motion capture system with markers, suggesting a high agreement. Moreover, the intra-trial reliability of each system was excellent, indicating high reproducibility. Therefore, the novel multi-view image-based motion analysis system may be a useful measurement tool to evaluate the intervention effects during the rehabilitation process and monitor the sports performance of athletes in sports training facilities and sports fields.
